# Comparison of Fracture Strength of Milled and 3D-Printed Crown Materials According to Occlusal Thickness

**DOI:** 10.3390/ma17184645

**Published:** 2024-09-22

**Authors:** Yeseul Park, Jimin Kim, You-Jung Kang, Eun-Young Shim, Jee-Hwan Kim

**Affiliations:** 1 Department of Prosthodontics, Oral Science Research Center, College of Dentistry, Yonsei University, Seoul 03722, Republic of Korea; yeseul189@yuhs.ac (Y.P.); jm321@yuhs.ac (J.K.); eun3690@gmail.com (E.-Y.S.); 2 Department of Prosthodontics, College of Dentistry, Yonsei University, Seoul 03722, Republic of Korea; kyj1219@yuhs.ac

**Keywords:** CAD/CAM, 3D-printed resin, milled hybrid ceramic, permanent prosthesis, fracture strength, Vickers hardness

## Abstract

This study aimed to measure the fracture strengths and hardness of final restorative milled and 3D-printed materials and evaluate the appropriate crown thickness for their clinical use for permanent prosthesis. One type of milled material (group M) and two types of 3D-printed materials (groups P1 and P2) were used. Their crown thickness was set to 0.5, 1.0, and 1.5 mm for each group, and the fracture strength was measured. Vickers hardness was measured and analyzed to confirm the hardness of each material. Scanning electron microscopy was taken to observe the surface changes of the 3D-printed materials under loads of 900 and 1500 N. With increased thickness, the fracture strength significantly increased for group M but significantly decreased for group P1. For group P2, the fracture strengths for the thicknesses of 0.5 mm and 1.5 mm significantly differed, but that for 1.0 mm did not differ from those for other thicknesses. The hardness of group M was significantly higher than that of groups P1 and P2. For all thicknesses, the fracture strength was higher than the average occlusal force for all materials; however, an appropriate crown thickness is required depending on the material and component.

## 1. Introduction

Advancements in digital technology have led to revolutionary changes in the field of dentistry. Particularly, the introduction of computer-aided design/computer-aided manufacturing (CAD/CAM) systems and 3D printing technology has greatly improved efficiency and accuracy in various fields, including diagnosis, treatment planning, and prosthesis manufacturing, and is also rapidly advancing in dental material development and manufacturing. In 1986, Charles (Chuck) Hull introduced the first stereolithography apparatus (SLA) type three-dimensional (3D) printing technology, and this technology has been applied to various fields, including medicine [1,2]. These technologies are currently a critical part of dental care, offering several advantages, including reduced treatment times and increased patient satisfaction through customized treatment. Various studies are being conducted, including the creation of final prostheses using digital technologies to continue to advance the digital field of dentistry [1,3,4,5,6].

Various materials, such as resin, ceramic, and zirconia, have been developed as final prosthetic materials for CAD/CAM, and hybrid materials, including polymer-infiltrated ceramic network, resin nano-ceramic, and zirconia-reinforced lithium silicate, have been introduced [7]. Compared with ceramic materials, CAD/CAM composites have the advantage of reducing the thickness of the restoration and their relatively low hardness and stiffness, which reduces wear on the natural tooth against which they are placed [7]. CAD/CAM materials are also easier to fabricate and remake using stored digital data and exhibit less cracking and chipping during fabrication and better marginal adaptation [8].

Considering methods for the manufacture of digital prostheses, the milling method can yield materials with high strength and surface roughness, although the results may vary depending on equipment performance and consumables, and this method has the disadvantage of lower fine part reproducibility than the 3D printing method [9]. Conversely, the 3D printing method is associated with limitations such as high initial cost due to equipment purchase and low marginal fit when compared with milling materials [1]. In terms of advantages, the 3D printing method can yield a pattern with a certain thickness and increase the reproducibility of fine details, as well as save time, cost, and materials when compared with the milling method. Hence, the 3D printing method is actively employed in various fields, including the dental fields of prosthetics, implants, reconstruction, and orthodontics, and is gradually replacing the existing method [10].

As CAD/CAM materials, 3D printing resins mainly include a composite resin polymer matrix and ceramic base filler particles and exhibit different properties depending on their composition. The properties of the material can be improved by increasing the filler content contained in the material, changing the particle size and shape, changing the matrix composition, and using a polymerization method [11].

The 3D-printed resins need mechanical properties that can withstand intraoral wear and tears to be used as the final dental prosthesis. CAD/CAM composite crowns can sufficiently withstand occlusal forces, have fewer catastrophic failures, chipping, and cracking, and are easy to repair [12]. Additionally, 3D-printed resin crowns have excellent physical properties and high fracture resistance [13]. Temporary restorations made using 3D printing methods have shown better marginal and internal adaptations than those produced using conventional or CAD/CAM milling methods [14,15,16].

Several studies have been conducted to evaluate the physical properties of 3D-printed resins. However, most are laboratory-level studies using standard blocks (ISO 10477 [17]) of rectangular or cylindrical shapes, excluding many variables that occur in clinical practice [18,19,20]. For other materials, some studies have suggested appropriate thicknesses depending on the physical properties of the clinically shaped crowns. For zirconia crowns, which are currently conventionally fabricated, a thickness of 1.0–1.5 mm in the occlusal plane is recommended [21], whereas other studies have suggested that CAD/CAM-fabricated crowns can withstand sufficient loads at a thickness of 0.5 mm [12]. However, research on the optimal thickness of final restorations fabricated using 3D printing is lacking. Therefore, further research is required on the physical properties of CAD/CAM materials using prosthetic fabrication methods in clinical practice.

In this study, the fracture strength and hardness of 3D-printed resins for different types of final restorations were measured in vitro as a function of the occlusal plane thickness to determine their potential as permanent prosthetic materials and the optimal thickness for their clinical application for different types of final restorations. The null hypothesis of this study was that the fracture strength of the 3D-printed resin does not differ from that of the milled hybrid ceramic.

## 2. Materials and Methods

**Figure 1:**
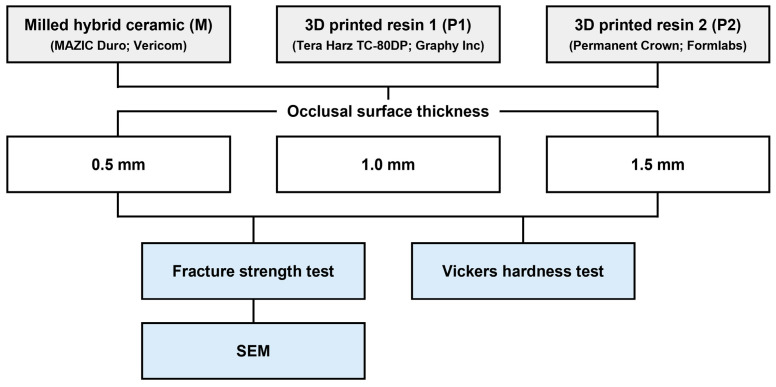
Schematic illustrating the experimental process.

Figure 1 presents the flow of the experimental procedure.

### 2.1. Materials

In this study, a milling block material of nanohybrid ceramic, composed of ceramic and high-density resin matrix (MAZIC Duro, Vericom, Chuncheon, Republic of Korea), was used as the control group. Two 3D-printed resins, 1 (Tera Harz TC-80DP, Graphy Inc., Seoul, Republic of Korea) and 2 (Permanent Crown, Formlabs, Somerville, MA, USA), were used as the experimental groups (Table 1).

### 2.2. Sample Preparation

**Figure 2:**
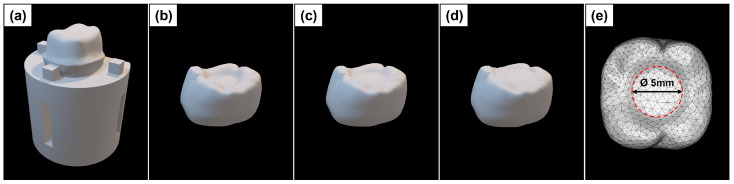
Prepared teeth metal dies and crowns according to occlusal thickness designed with a CAD software program. (**a**) Prepared teeth metal die; (**b**) Crown of 0.5 mm thickness; (**c**) Crown of 1.0 mm thickness; (**d**) Crown of 1.5 mm thickness; and (**e**) The geometry model of the crown.

The G*Power software (version 3.1.9.4) was employed to determine the sample size required for this study. Twelve samples per group were satisfactory for achieving a 90% study power with the alpha probability error and effect size set to 0.05 and 0.348, respectively, for a total sample size of 108 specimens. The specimens for fracture strength tests were prepared as follows: A tooth model corresponding to the first molar of the left maxillary was prepared using a standard dentiform (ANA-4 ZP, Frasaco GmbH, Tettnang, Germany). The tooth model was prepared by a prosthodontist (J. H. K.) with more than 20 years of experience in crown preparation. The prepared tooth model was scanned using a dental scanner (T500, Medit, Seoul, Republic of Korea) and imported into a CAD software program (Rhinoceros 5.0, Robert McNeel & Associates, Seattle, WA, USA) (Figure 2a). After designing the die replica in the program, 12 copies were printed using a standard tessellation language (STL) file with titanium alloy 3D printing material (Ti64 Grade 23, GE Additive, West Chester Township, OH, USA) on a metal 3D printer (Rainbow Metal Printer, Dentium Co., Suwon, Republic of Korea). The powder was sprayed onto the printed metal die for scanning using an intraoral scanner (TRIOS3, 3Shape A/S, Copenhagen, Denmark).

The STL file of the scanned die was loaded into a CAD software program (Exocad DentalCAD 2.2, Exocad GmbH, Darmstadt, Germany) to design a resin crown. The thickness of the wall (mm) and margin (mm) of the resin crown were set to be the same, and the thickness of the occlusal surface was set to 0.5 mm, 1.0 mm, and 1.5 mm (Figure 2b, Figure 2c, and Figure 2d, respectively). The cement space between the resin crown and the metal die was set to 70 μm, and 12 crown specimens were fabricated for each group.

The specimens for the Vickers hardness measurement were designed to be rectangular with dimensions of 15 mm × 15 mm × 5 mm using a CAD software program, an open source program for 3D design (TinkerCAD [https://www.tinkercad.com/], Autodesk Inc., San Rafael, CA, USA). Three specimens from each group were fabricated according to the manufacturer’s instructions.

The milled hybrid ceramic blocks were fabricated as follows: first, crowns of three thicknesses and rectangular prism specimenswere milled using a disc-type composite resin block (Mazic Duro; Vericom) on a milling machine (MC4D-001, Dental Plus, Seongnam, Republic of Korea). The milled specimens were cleaned for 5 min using an ultrasonic cleaning system (Fine Sonic E20, Vericom, Chuncheon, Republic of Korea).

The 3D-printed resin 1 was fabricated as follows: the STL files of crowns and rectangular prism specimens designed based on thickness were imported into the 3D printer software (UNIZ Maker 1.5.4.35, UniZ Technology, San Diego, CA, USA). The specimen designs were arranged on the platform; the supports were attached perpendicularly to the occlusal plane and then printed on a DLP-type 3D printer (Sprint Ray Pro 95, SprintRay, Los Angeles, CA, USA) using a liquid-curable 3D printing resin (Tera Harz TC-80DP; Graphy Inc.). The printed specimens were cleaned with 95% isopropyl alcohol for 5 min in an ultrasonic cleaner (Shinhan 200H 3 L, Shinhan-sonic, Incheon, Republic of Korea) and then washed with a light-emitting diode (wavelength 405 nm and light intensity of 400 mW/cm^2^) according to the manufacturer’s instructions (LED). Subsequently, the printed specimens were post-cured for 30 min in a polymerization device (Cure-M 102H, Sona Global Co., Ltd., Seoul, Republic of Korea) and stored at room temperature (22 ± 1 °C).

The 3D-printed resin 2 was fabricated similarly to 3D-printed resin 1 but with a different device following the manufacturer’s instructions. First, the design STL file was imported using 3D printer software (PreForm 3.27.0; Formlabs) and then printed on a stereolithography 3D printer (Form 3; Formlabs) using a 3D printing resin (Permanent crown; Formlabs). After washing and drying in the same manner, the samples were cured for 30 min in a curing device (Formcure; Formlabs).

The milled hybrid ceramic was named group M, 3D-printed resin 1 was named group P1, and 3D-printed resin 2 was named group P2. They were marked as X–Y (such as M-0.5), where X and Y represent the group name and occlusal thickness, respectively.

### 2.3. Fracture Strength Test

For the fracture strength test, the crowns were cleaned in an ultrasonic cleaner (SD-120H, Sungdong Ultrasonic Co., Seoul, Republic of Korea) and distilled water for 3 min and dried; an adhesive bonding agent (single bond universal [SBU], 3M ESPE, St. Paul, MN, USA) was then applied to the inner surface. Subsequently, the air was blown for 5 s and the samples were cured for 10 s. The inner surface-treated crowns were bonded to a metal die using self-adhesive resin cement (Rely X U200, 3M ESPE, St. Paul, MN, USA). All crowns were fully fitted onto the abutment using finger pressure, adhesive-cured to remove excess cement, and hardened for 20 s. The prepared specimens were fixed on a metal jig perpendicular to the crowns, and the fracture strength was measured using a universal testing machine (UTM; 3366 Series, Instron Engineering, Norwood, MA, USA). A 3 mm stainless steel ball (stainless-steel 304, Elim Co., Yang-Ju, Republic of Korea) was placed to contact the crown center in the vertical axis, and the specimen was tested until fracture with the crosshead speed set to 1 mm/min. The measured fracture load was recorded in Newton (N), and 36 specimens from all groups were measured repeatedly using the same method. Compared with the other groups where fracture values were recorded, P1-1.0 did not fracture during the UTM experiment; therefore, it was recorded as an extreme strength value on the load-displacement graph.

### 2.4. Vickers Hardness Test

The top surface of each specimen was polished using 1000-grit silicon carbon abrasive paper at 300 rpm under tap water irrigation to create a specimen with a uniform surface and remove contaminants. Nine specimens (three from each material) were produced and classified into three groups. The experiment was conducted using a Vickers microhardness meter (MH3, Mekton, Bursa, Turkey) with a load (500 g) and a dwell time (15 s) to form the indentation. The measurements were repeated five times per specimen, and the length of the indentation diagonal (*d*) was observed at 100× magnification using an optical microscope equipped with a micro-Vickers hardness tester after the indentation was formed.

Vickers hardness (*HV*) was calculated as follows:

$$HV=\kappa \frac{P}{d^{2}}$$

*κ*: 1.8544

*P*: Load (kgf)

*d*: Mean diagonal of indentation (mm)

### 2.5. Scanning Electron Microscopy (SEM)

For P1-1.0, which showed little fracturing during the fracture test, two specimens were prepared separately for SEM (S-300N, Hitachi, Tokyo, Japan) at loads of 900 and 1500 N during the fracture test. Each specimen was coated with 100 nm of platinum using an ion sputter coater (E-1010, Hitachi, Tokyo, Japan), and images were captured at 18× and 300× magnification to observe the morphological microstructure of the outer surfaces of the central part of the crown where the load was applied. The group P2 specimen was also photographed using the same method for comparison with the same 3D printing material.

### 2.6. Statistical Analysis

All measured results were analyzed separately for fracture strength and Vickers hardness using IBM SPSS Statistics for Windows, version 26.0 (IBM Corp., Armonk, NY, USA). All data were analyzed for normality using the Kolmogorov–Smirnov test. After verifying the normality of the data for the fracture strength test using two-way analysis of variance (ANOVA), Bonferroni’s multiple comparison test was applied to compare the differences in each group and thickness. For the Vickers hardness test, the Kruskal–Wallis test was used to analyze the effects of the materials on the surface hardness and the differences. The significance level was set at 95% (α = 0.05).

## 3. Results

### 3.1. Fracture Strength Test

**Figure 3:**
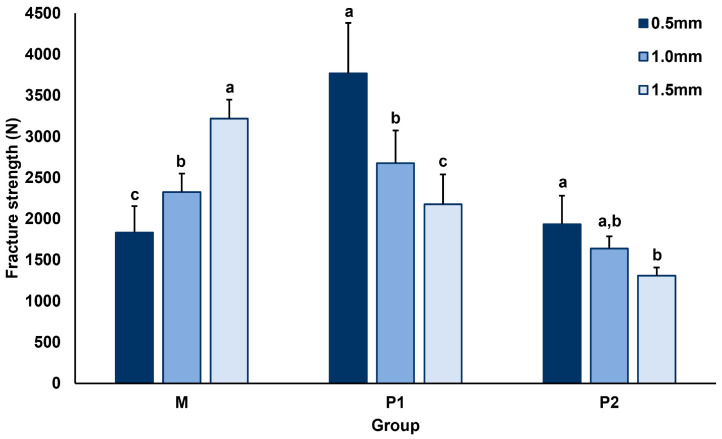
Mean and standard deviation (SD) of the fracture strength between groups. M, milled hybrid ceramic; P1, 3D-printed resin 1; P2, 3D-printed resin 2. Lowercase letters indicate differences between crown thickness in materials.

The fracture strength test recorded the load at which the crown of each group was fractured (N). Displacement-load graphs for the fracture strength of each material are presented in Supplementary Figures S1–S3. The average fracture strengths and significant differences among the three groups according to thickness are presented in Table 2 and Figure 3. A two-way ANOVA was conducted to verify the main and interaction effects of material and thickness on the fracture strength. The results showed that the main effects of material (F = 127.538, *p* < 0.001) and thickness (F = 8.766, *p* < 0.001) on fracture strength were significant. Additionally, the interaction effect between material and thickness on fracture strength was also significant (F = 61.526, *p* < 0.001).

In the post-hoc test, the fracture strength according to the thickness increased as the thickness increased in group M and decreased as the thickness increased in groups P1 and P2. Additionally, fracture strength was significantly lower in group P2 than in the other groups, and P1-0.5 had the highest fracture strength. In group M, M-0.5 showed the lowest fracture strength at 1834.6 (±323.3) N; as the thickness increased, the fracture strength also increased, showing a significant difference from M-1.0 and M-1. However, in group P1, P1-0.5 showed the highest fracture strength among all groups at 3769.7 (±613.9) N. As the thickness increased, the fracture strength decreased, and a significant difference was observed between P1-1.0 and P1-1.5. P2-0.5 showed the highest strength in group P2 at 1932.7 (±347.6) N; similarly to group P1, as the thickness increased, the fracture strength decreased. P2-0.5 and P2-1.5 showed a significant difference; however, P2-1.0 showed no significant differences.

In a comparison of fracture strength by material, P1-0.5 was 3769.7 (±613.9) N, which was significantly higher than that with the same thickness in other groups. At 1.0 and 1.5 mm thickness, all three groups showed significant differences. P1-1.0 showed the significantly highest value of 2677.3 (±398.2) N, and M-1.5 had the highest value of 3218.8 (±228.7) N at their respective thicknesses.

### 3.2. Vickers Hardness Test

The mean value, standard deviation, and statistical analysis of the Vickers hardness in each group are presented in Table 3. The effect of the prosthesis thickness on the surface hardness was significantly different among the three groups (*p* < 0.001). The surface hardness of group M was 111.18 (±9.90) HV, which was higher than that of the other groups, showing a significant difference. Group P2 showed a significantly higher hardness of 33.39 (±2.90) HV than group P1 21.64 (±1.33) HV.

### 3.3. SEM

**Figure 4:**
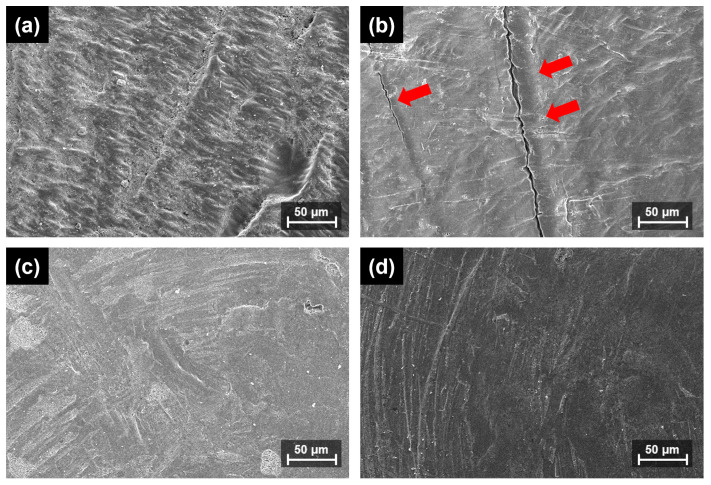
High-magnification (×300) Scanning electron microscopy images of both types of 3D-printed resin crowns for load area on the outer surface: (**a**) Load area of P1-1.0 with 900 N force applied; (**b**) Load area of P1-1.0 with 1500 N force applied; (**c**) Load area of P2-1.0 with 900 N force applied; and (**d**) Load area of P2-1.0 with 1500 N force applied. The red arrows indicate microcracks on the surface. Only the P1-1.0 group with 1500 N force applied shows microcracks.

Compared with groups M and P2, where none of the crowns remained attached to the metal die after the fracture strength test, in group P1, the crowns remained attached to the metal die whether a fracture occurred or not. For comparison with P1-1.0, where 10 of 12 crowns did not fracture upon visual observation, SEM images of specimens subjected to the same load (N) in the same 3D-printed resin, P2-1.0, are presented. Supplementary Figure S4 presents low-magnification images, whereas Figure 4 presents high-magnification images.

After applying a load of 900 N in P1-1.0, no crack lines or fractured areas were observed by the naked eye; the same was observed in the SEM images. At 1500 N, no abnormalities were observed with the naked eye. However, when observed with SEM, crack lines were observed on the outer surface, and the inner cement was broken. When the same 3D-printed resin material, P2-1.0, was examined using SEM after applying the same load pressure, no crack lines or fractured parts were observed.

## 4. Discussion

In this study, the fracture resistance of one milled hybrid ceramic and two 3D-printed resin crowns used as permanent prosthesis materials was evaluated by measuring the fracture strength according to the thickness of the posterior occlusal surface through in vitro experiments. Comparing the fracture strength according to the crown thickness and material in this study revealed an increase in the fracture strength of the milled hybrid ceramic as the thickness increased from 0.5 mm to 1.5 mm. However, the fracture strength decreased as the thickness increased in the two groups of 3D-printed resins. Therefore, the null hypothesis of this study was rejected.

The lowest fracture strength values for the mechanical strength of the materials tested in this study were 1307.5 N, 2176.8 N, and 1834.6 N for P2-1.5, P1-1.5, and M-0.5, respectively, which is higher than the average occlusal bite force of approximately 500 N and the maximum bite force of approximately 900 N previously reported [22]. Previous studies examining the fracture resistance of temporary crowns fabricated by 3D printing and milling methods reported that milled molar crowns (1817.50 N) had a higher fracture strength than 3D-printed crowns (1189.50 N). However, no significant difference was observed in the incisors, and all were reported to be clinically safe to use with sufficient strength to withstand masticatory pressure [23]. Furthermore, the average fracture resistance of the milled premolars was 1890 N in a study on the fracture resistance of milled zirconia and 3D-printed crowns, whereas that of the 3D-printed group was 1658 N. The 3D-printed crown had a lower value; however, it was considered clinically acceptable because it was higher than the normal masticatory force [24]. Therefore, this study indicates that the mechanical strength of all materials tested can adequately withstand the vertical pressure caused by the actual occlusion. The strength and surface properties of 3D-printed resin crowns for first molars have been examined, and 3D-printed resin crowns were proposed as a treatment option for pediatric and adolescent patients [25]. Likewise, the average occlusal force of patients varies depending on age and sex, and the properties, strength, and thickness of the prosthesis required can vary depending on the type of opposing teeth. In addition, the required esthetic properties may vary depending on the thickness of the prosthesis. Therefore, the results of this study may be valuable in selecting 3D-printed resin crowns with appropriate strength and thickness for various patients.

Both 3D-printed resins showed a tendency for the fracture strength to decrease as the thickness increased, and group P1 exhibited a significantly higher fracture strength than group P2 at all three thicknesses. Consistent results have been reported by a previous study evaluating the fracture strength using the same material as group P1 [25,26]. Group P1 has a high mechanical strength owing to the large molecular weight of the composed oligomer, although it is flexible due to the low cross-linking density accompanied by high deformation characteristics. Based on these properties, fracture strength may decrease as the occlusal thickness increases [26]. The fracture strength of the material of hybrid ceramics using the milling method increased as the thickness increased. This is consistent with previous reports [27,28,29].

In this study, the use of titanium alloy as the dies resulted in high average fracture strength values. In a previous study using a metal die similar to this study, the average fracture load values of CAD/CAM ceramic materials ranged from 1446 to 2437 N [28], revealing high fracture strength. Metal dies can facilitate standardized preparation, ensure the die will not break or be damaged during testing, and maintain the identical physical quality of materials [30,31]. Although metal dies cannot reproduce the actual force distribution that may occur in crowns cemented to natural teeth, using natural teeth as dies may increase the variability of the results, given that the uniformity of collection, storage, and preparation cannot be controlled [27]. In the future, it is necessary to conduct studies using composite materials with a modulus similar to dentin.

Surface-hardness tests are useful for evaluating the wear resistance of a material [32]. Here, the surface hardness values showed significant differences between the milled and 3D-printed materials. Group M had a value of 111.18 HV; however, groups P1 and P2 had 21.64 HV and 33.39 HV, respectively, showing hardness values that significantly differed from those of group M by approximately 89.54 HV and 77.79 HV, respectively. This is believed to be due to the difference in physical properties resulting from the different material compositions of the milling and 3D printing methods [33,34]. In previous studies, the surface hardness of milled and 3D-printed materials was observed, and 3D-printed specimens had lower surface hardness than milled specimens; the filler content in 3D-printed materials ranged from 3 to 50 wt%, compared to the approximately 70 wt% filler content observed in milled materials, and the filler content appeared to affect the hardness [35]. Additionally, a study comparing the mechanical properties of 3D-printed resins concluded that resins with lower hardness values than dentin (approximately 65 HV) were due to a low filler content [36,37]. Compared with the specimen materials in this study, the milled hybrid ceramic had a hardness higher than that of dentin, which was approximately 65 HV. The 3D-printed resin did not attain the hardness of dentin for either material. This was probably because group P1 contained no filler compared with group M’s filler content of approximately 76 wt%, and group P2 contained only a low filler content of approximately 30–50 wt%.

After the fracture experiment, most of the specimens in groups M and P2 showed the same fracture behavior of breaking into two or more pieces and then separating from the metal die, whereas group P1 showed a sagging fracture behavior; particularly, 10 specimens in P1-1.0 did not fracture but were deformed and separated from the metal die. Therefore, the fracture strength of P1-1.0 could not be measured because no actual fracture occurred in the UTM test; thus, the ultimate strength was calculated from the load-displacement graph. P1-1.0 consistently experienced necking after reaching its ultimate strength [38,39]. SEM images were obtained for crack detection and surface analysis at loads before reaching the fracture strength of P1-1.0 presented in this study. The loads were set at 900 N, which is the maximum occlusal pressure degree, and 1500 N, which is below the average fracture strength value of P2-1.0 [22,24]. When P1-1.0 was observed with SEM, no fracture occurred at 900 N, and no fracture occurred at 1500 N; however, deformation or microcracks were confirmed. Conversely, P2-1.0 did not fracture or crack at either 900 N or 1500 N. This suggests that P1-1.0 cracked before the fracture loads measured in this study were reached. The development of cracks, deformation, and sagging of crowns without complete fracture was likely due to the flexibility of the material. These microcracks may lead to future fractures and secondary caries, which may negatively affect tooth prognosis [40,41,42].

Previous studies on the material properties of 3D-printed and hybrid prosthetics have concluded that the bending strength can be higher depending on the type and content of the resin, in addition to the filler content. At similar filler contents, materials containing Bis-GMA have a higher strength, modulus, and hardness than those with UDMA [35]. Furthermore, in other studies that tested the influence of the resin matrix, viscosity decreased in the following order: UDMA > Bis-EMA > Bis-GMA + TEGDMA. A lower viscosity enables higher conversion rates, which in turn allows for more fillers [43,44]. Bis-GMA has a high molecular weight and a low double-bond concentration; therefore, it exhibits low polymerization shrinkage and excellent durability after curing. Bis-EMA has a high molecular weight but has no hydroxyl groups; thus, it has low viscosity and high conversion, which can improve its physical properties. UDMA has high flexural strength, elastic modulus, and strength; however, its high viscosity limits the amount of filler it can contain [34,44,45,46,47]. Group P1 is a UDMA oligomer that contains no fillers. Group P2 contains inorganic fillers, whereas group M contains ceramic fillers. In P1-0.5, the fracture strength was the highest owing to the UDMA characteristics; however, it appeared to have fractured due to the low surface hardness, high risk of wear, and insufficient thickness to withstand the load. In contrast, P1-1.0 exhibited microcracks and deformation before fracture, and in P1-1.5, all materials were fractured at a load exceeding 2000 N, with almost no material sagging. Therefore, when using the material in group P1 to fabricate a crown, a thickness of at least 1.5 mm or more on the occlusal surface should be appropriate; however, for this material to be used as a final prosthesis, improving its physical properties by adding fillers and improving the resin matrix is required [34,47]. In particular, in the case of 3D printing, the mechanical properties of the 3D-printed resin may be reduced owing to poor adhesion between adjacent layers at the top of the specimen due to the printing layering method. Therefore, it is crucial to select an appropriate thickness for the occlusal surface when manufacturing a crown using the 3D printing method [48]. In group P2, P2-0.5 had the highest strength; however, no significant difference was observed in fracture strength from P2-1.0, and P2-1.5 had the lowest fracture strength. Because group P2 has a low surface hardness, the risk of wear may be high (0.5 mm); therefore, a thickness of at least 1.0 mm on the occlusal surface appears appropriate for making crowns using group P2 material. In group P2, if the filler content is increased, better mechanical properties [47]. In group M, the strength increased as the thickness increased, the surface hardness was high, and the fracture strength was higher than the average occlusal force, even at 0.5 mm. Therefore, a thickness of at least 0.5 mm or more on the occlusal surface is required when making a crown.

This study had some limitations. It compared and evaluated the fracture resistance according to the occlusal surface thickness of posterior crowns manufactured using milling and 3D printing materials. The axial walls of the crowns were designed identically for the experiment. Additionally, under the experimental conditions of this study, only vertical loads were applied to the crowns; therefore, variables such as saliva, food, and lateral occlusal force that may occur in the actual oral cavity were excluded, limiting the determination of an accurate clinical prognosis. Therefore, further research on other mechanical properties, such as fatigue strength, solubility, wear, and bonding strength, by reproducing the oral environment, including clinical research on long-term stability, should be conducted. Furthermore, research is required to strengthen the physical properties by including fillers in 3D-printed resin materials or by increasing their amount. If these additional experiments are conducted and various evaluations of 3D-printed resin crowns and material improvements are made, 3D printing crown production could become an effective treatment option for prosthetic restorations.

## 5. Conclusions

The following conclusions are derived from the study:

1. The fracture strength of the milled materials increased with increasing crown thickness, whereas that of 3D-printed materials decreased with increasing crown thickness. However, all groups had higher fracture strengths than the average occlusal force.
2. The values of Vickers hardness were 111.18 (±9.90) HV, 33.39 (±2.90) HV, and 21.64 (±1.33) HV for groups M, P2, and P1, respectively. Groups M and P2, which contained fillers, exhibited significantly higher hardness than group P1.
3. The appropriate crown thickness required depends on the material and composition of the restoration.

## Figures and Tables

**Table 1:** Compositions of the materials used in this study.

Product Name	Composition	Manufacturer
MAZIC Duro (A2)	20% resin matrix (Bis-GMA, TEGDMA), 76% nanoparticle-sized ceramic fillers (zirconia, silica, barium aluminosilicate)	Vericom, Chuncheon, Republic of Korea
Tera Harz TC-80DP (A2)	70% Urethane dimethacrylate oligomer, 30% resin mixture (mono-, di- acrylate), Phenylbis (2,4,6-trimethylbenzoyl) phosphine oxide (BAPO, photoinitiator), pigment	Graphy Inc., Seoul, Republic of Korea
Permanent Crown (A2)	Esterification products of 4,4′-isopropylidiphenol, ethoxylated and 2-methylprop-2enoic acid; ethoxylated bisphenol-A dimethacrylate (Bis-EMA, methacrylate polymer), silanized dental glass, methyl benzoylformate, diphenyl (2,4,6-trimethylbenzoyl) phosphine oxide (TPO, photoinitiator), 30–50 wt%—inorganic fillers (particle size 0.7 μm)	Formlabs, Somerville, MA, USA

**Table 2:** Mean and standard deviation (SD) of the fracture strength for each group (Mean ± SD).

Occlusal Thickness (mm)	Group		
	M	P1	P2
0.5	1834.6 ± 323.3 ^b^	3769.7 ± 613.9 ^a^	1932.7 ± 347.6 ^b^
1.0	2326.3 ± 225.0 ^ab^	2677.3 ± 398.2 ^a^	1638.0 ± 148.1 ^c^
1.5	3218.8 ± 228.7 ^a^	2176.8 ± 362.0 ^b^	1307.5 ± 102.0 ^c^

M, milled hybrid ceramic; P1, 3D-printed resin 1; P2, 3D-printed resin 2. Lowercase letters indicate differences between materials in crown thickness.

**Table 3:** Mean and standard deviation (SD) of Vickers hardness for each crown material (Mean ± SD).

M	P1	P2
111.18 ± 9.90 ^a^	21.64 ± 1.33 ^c^	33.39 ± 2.90 ^b^

M, milled hybrid ceramic; P1, 3D-printed resin 1; P2, 3D-printed resin 2. Lowercase letters indicate differences between crown materials.

## Data Availability

The original contributions presented in the study are included in the article and Supplementary Materials, further inquiries can be directed to the corresponding author.

## Supplementary Materials

The following supporting information can be downloaded at: https://www.mdpi.com/article/10.3390/ma17184645/s1. Figure S1: The load-displacement graph of group M: (a) M-0.5; (b) M-1.0; and (c) M-1.5; Figure S2: The load-displacement graph of group P1: (a) P1-0.5; (b) P1-1.0; and (c) P1-1.5; Figure S3: The load-displacement graph of group P2: (a) P2-0.5; (b) P2-1.0; and (c) P2-1.5; Figure S4: Low-magnification (×18) SEM images of both types of 3D printing resin crowns for load area on the outer surface: (a) Load area of P1-1.0 with 900 N force applied; (b) Load area of P1-1.0 with 1500 N force applied; (c) Load area of P2-1.0 with 900 N force applied; and (d) Load area of P2-1.0 with 1500 N force applied. The red dotted circles indicate the load area.
