# Supplementary material for: Comparison of Fracture Strength of Milled and 3D-Printed Crown Materials According to Occlusal Thickness
Source: Materials (Basel). 2024 Sep 22;17(18):4645. doi: 10.3390/ma17184645 (PMC11433717; doi:10.3390/ma17184645)
Supplement: Supplementary file 1 [file materials-17-04645-s001.zip › materials-3208392-supplementary.pdf]

Supplementary Materials

Comparison of Fracture Strength of Milled and 3D-Printed Crown Materials According to Occlusal Thickness

Yeseul Park [1,†], Jimin Kim [1,†], You-Jung Kang [2], Eun-Young Shim [1] and Jee-Hwan Kim [2,*]

[1] Department of Prosthodontics, Oral Science Research Center, College of Dentistry, Yonsei University,
Seoul 03722, Republic of Korea; yeseul189@yuhs.ac (Y.P.); jm321@yuhs.ac (J.K.); eun3690@gmail.com (E.-Y.S.)

[2] Department of Prosthodontics, College of Dentistry, Yonsei University, Seoul 03722, Republic of Korea; kyj1219@yuhs.ac

* Correspondence: jee917@yuhs.ac; Tel.: +82-2-2228-3161; Fax: +82-2-312-3598

† These authors contributed equally to this work.

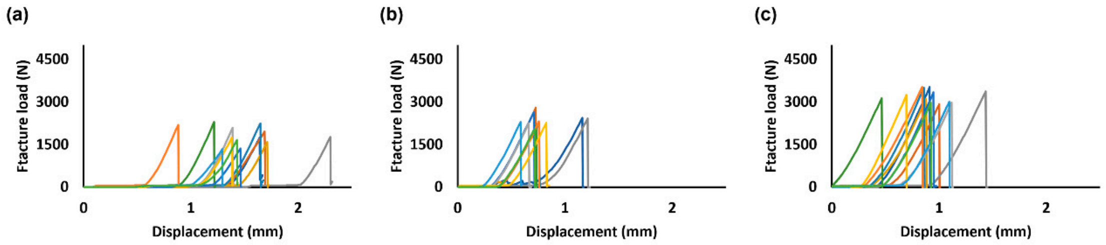

Supplementary Figure S1. The load-displacement graph of group M: (a) M-0.5; (b) M-1.0; and (c) M-1.5.

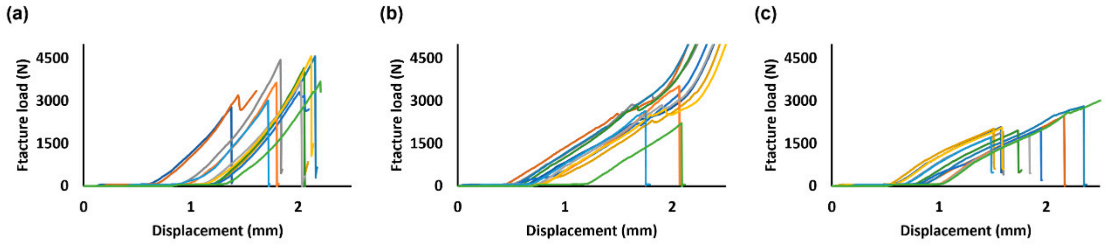

Supplementary Figure S2. The load-displacement graph of group P1: (a) P1-0.5; (b) P1-1.0; and (c) P1-1.5.

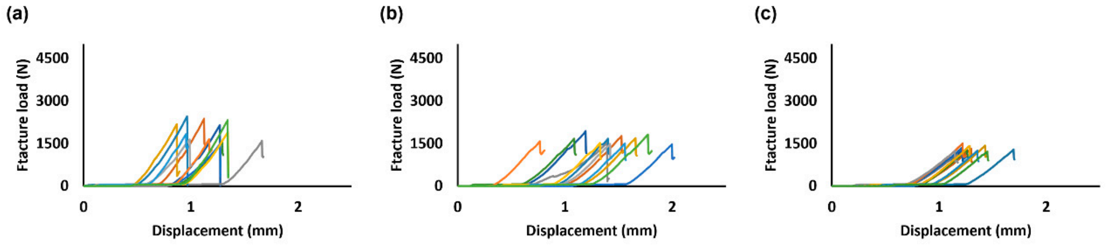

Supplementary Figure S3. The load-displacement graph of group P2: (a) P2-0.5; (b) P2-1.0; and (c) P2-1.5.

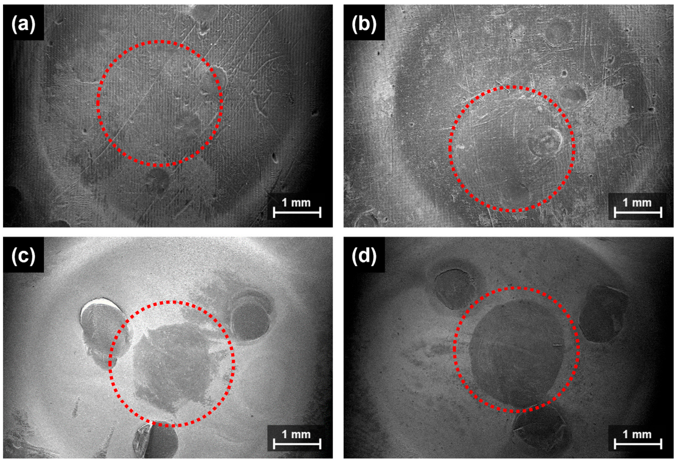

Supplementary Figure S4. Low-magnification (×18) SEM images of both types of 3D printing resin crowns for load area on the outer surface: (a) Load area of P1-1.0 with 900 N force applied; (b) Load area of P1-1.0 with 1500 N force applied; (c) Load area of P2-1.0 with 900 N force applied; and (d) Load area of P2-1.0 with 1500 N force applied. The red dotted circles indicate the load area.
